# Palliative treatment of presacral recurrence of endometrial cancer using irreversible electroporation: a case report

**DOI:** 10.1186/1752-1947-7-128

**Published:** 2013-05-13

**Authors:** Christoph Niessen, Ernst-Michael Jung, Andreas G Schreyer, Walter A Wohlgemuth, Benedikt Trabold, Joachim Hahn, Michael Rechenmacher, Christian Stroszczynski, Philipp Wiggermann

**Affiliations:** 1 Department of Radiology, University Hospital Regensburg, Franz-Josef Strauss Allee 11, Regensburg, 93042, Germany; 2 Department of Anaesthesiology, University Hospital Regensburg, Franz-Josef Strauss Allee 11, Regensburg, 93042, Germany; 3 Department of Hematology and Oncology, University Hospital Regensburg, Franz-Josef Strauss Allee 11, Regensburg, 93042, Germany

## Abstract

**Introduction:**

Irreversible electroporation (IRE) is a new minimally invasive tumor ablation technique which induces irreversible disruption of cell membrane integrity by changing the transmembrane potential resulting in cell death. Irreversible electroporation is currently undergoing clinical investigation as local tumor therapy for malignant liver and lung lesions. This is the first case report to describe the successful palliative ablation of a presacral recurrence of an endometrial cancer to achieve locoregional tumor control and pain relief.

**Case presentation:**

A 56-year-old Caucasian woman was referred for interventional treatment of an advanced local recurrence of endometrial cancer (11.9 × 11.6 × 14.9cm) with infiltration of the sacral bone and nerve plexus. Due to the immediate proximity to the sacral plexus, the patient could neither undergo surgical therapy nor a second radiation therapy. Due to its ablation mechanism irreversible electroporation was deemed to be the best therapy option.

**Conclusion:**

We showed in this case that a large tumor mass adjacent to a bundle of neural structures, the sacral plexus, can be widely ablated by irreversible electroporation with only minor temporary impairment of the neural function, even though a large infiltrating tissue volume (941cm^3^) was ablated.

## Introduction

Irreversible electroporation (IRE) is a new non-thermal local ablative treatment procedure which induces the irreversible permeabilization of a membrane lipid bilayer by creation of nanopores resulting in cell death [1]. IRE is currently undergoing clinical investigation as a locally ablative tumor therapy for different organ systems, such as kidney, lung or liver lesions [2].

Endometrial cancer represents the seventh most frequent tumor worldwide in women with an annual incidence of 142,000 patients worldwide [3]. The standard therapy for endometrial cancer is total removal of the uterus, cervix, as much as the parametrial tissue as possible, and a wide margin of the vagina (Wertheim-Meigs operation). At advanced stage endometrial cancer adjuvant radiation is performed. Radiation is considered standard therapy of inoperable tumor stages [4].

This article reports the successful palliative ablation of a presacral recurrence of endometrial cancer as an individual palliative therapy trial in order to achieve locoregional tumor control and pain relief.

## Case presentation

A 56-year-old Caucasian woman was referred for treatment of a local recurrence of endometrial cancer with infiltration of her sacral bone and nerve plexus. After initial diagnosis of endometrial cancer in 2005 (pT1B N0 MX G1 L0 V0), a Wertheim-Meigs operation was performed with subsequent radiotherapy with the initial result of complete tumor remission. In 2009 the patient presented with histologically confirmed pulmonary metastases. Under anti-hormonal therapy (medroxyprogesterone acetate), again complete remission was achieved. At the start of 2012, 7 years after the initial diagnosis, the patient was admitted to our hospital due to a presacral recurrence of the endometrial cancer with infiltration and widespread destruction of the os sacrum.

**Figure 1:**
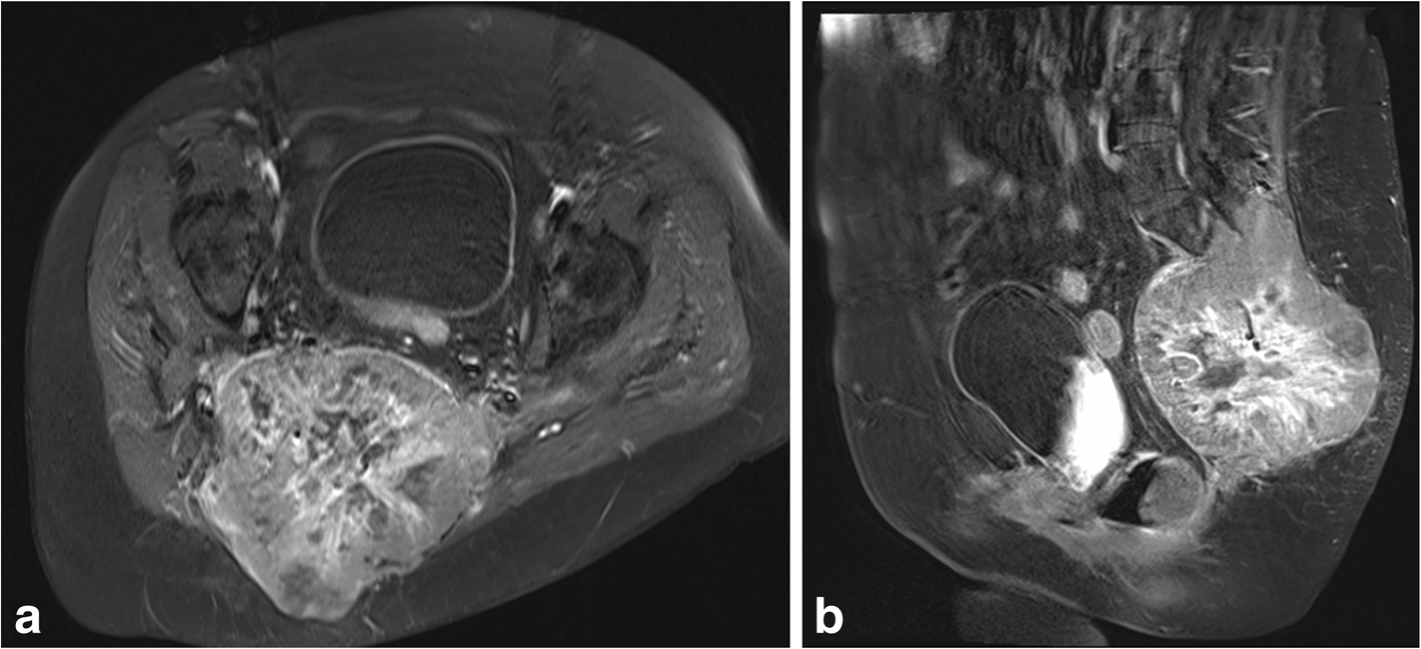
**Baseline postcontrast T1-weighted magnetic resonance images (a: axial plane; b: sagittal plane)**. Preinterventional images depicting the large (11.9 × 11.6 × 14.9 cm) viable tumor mass with infiltration and destruction of the os sacrum as well as infiltration of the sacral nerve plexus.

The patient complained of severe back pain and a marked pain spreading out to her right leg, suggestive of nerve infiltration. Magnetic resonance imaging (MRI) showed infiltration of the os sacrum and the sacral plexus. The tumor size was 11.9 × 11.6cm in axial section and 14.9cm in craniocaudal direction (Figure 1).

The patient’s case was discussed at the multidisciplinary tumor board for therapy options: due to the immediate proximity to the sacral plexus the patient could neither undergo surgical therapy nor a second radiation therapy because of the high risk of neural destruction potentially resulting in paralysis. Due to its ablation mechanism IRE was deemed to be the best therapy option for the patient as palliative disease control.

IRE uses a series of electrical pulses of microseconds to generate irreversible permeabilization of cell membranes and thereby induces cell death in the treated region. IRE seems to be highly effective in tissues with a high density of cell wall structures and less effective in tissues with a high concentration of collagenous and elastic fibers [5,6]. This – in contrast to thermal ablation techniques like radiofrequency ablation (RFA) or microwave ablation – potentially allows tumor cell ablation without concomitant destruction of connective tissue, blood vessels and nerves [7]. Due to this potentially selective cell ablation technique, IRE was offered as a palliative therapy option because it provided the opportunity of tumor mass reduction and decrease of tumor burden with reduced risk of impairment of the sacral plexus and surrounding blood vessels. The procedure with risks and benefits was discussed with the patient and informed consent was obtained.

**Figure 2:**
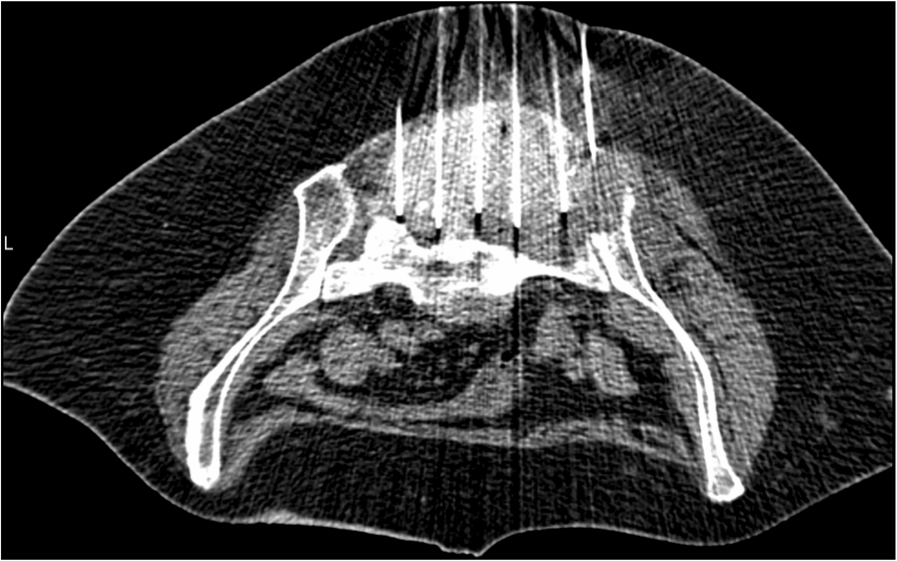
Intraprocedural computed tomography fluoroscopy image showing the six ablation electrodes in the tumor mass.

**Figure 3:**
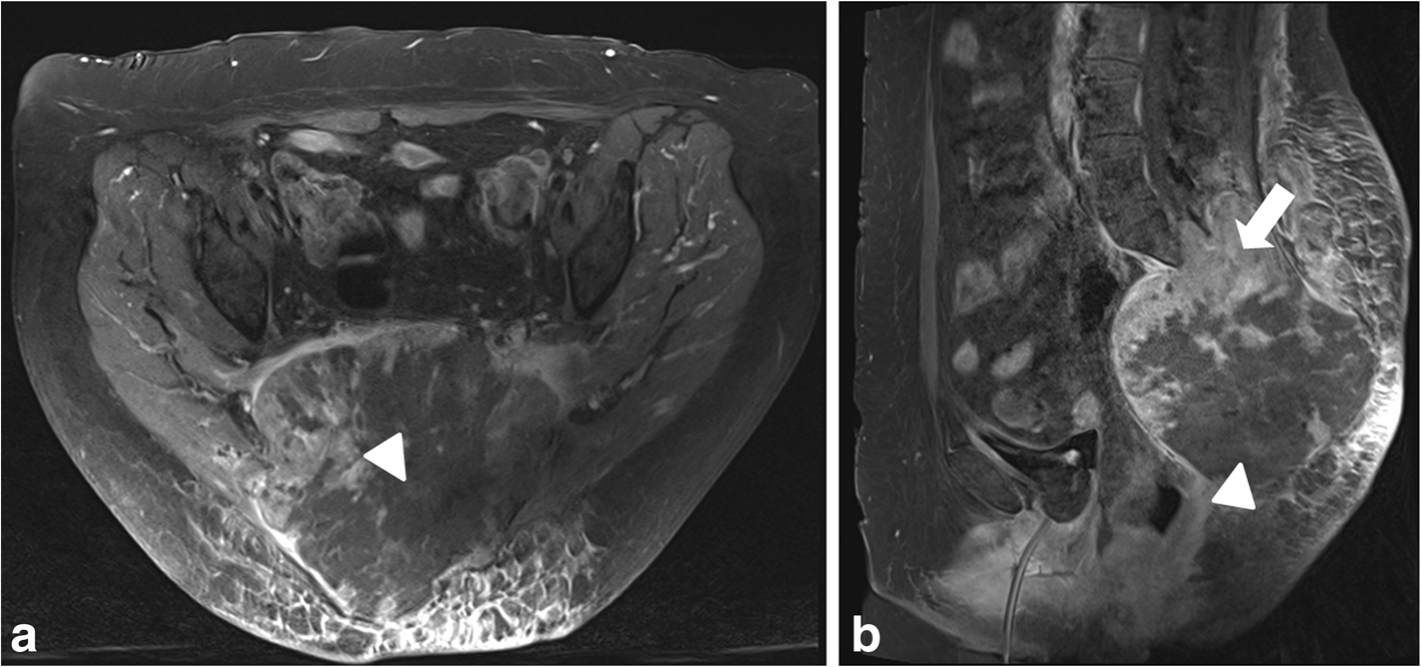
**Follow-up postcontrast T1-weighted magnetic resonance images at 24 hours after first intervention.** Viable enhancing tumor tissue (arrow) in the cranial and peripheral part of the lesion. Central parts of the tumor are necrotic (arrow heads). (**a**: axial plane, **b**: sagittal plane).

The patient was put under general anesthesia and neuromuscular blocking to prevent arrhythmia [8]. The procedure was performed using a commercially available IRE system. Due to the large tumor volume (941cm^3^) a total of six needles were placed into the target area (Figure 2). The percutaneous placement of the electrodes was guided by computed tomography (CT) fluoroscopy as well as ultrasound using a multifrequency probe (1 to 5MHz). As recommended by the manufacturer and recorded by the IRE generator the following parameters were used: number of electrodes: six; type of electrodes: monopolar; distance of electrodes: 0.7cm (minimum), 1.2cm (maximum); impulses per electrode: 70; voltage: 1100V (minimum), 3000V (maximum). A stepwise ablation procedure with multiple replacements of the electrodes was performed, starting from a caudal position and moving to cranial. The first ablation procedure covered about 40% of the tumor mass and was stopped after 8 hours. Follow-up imaging showed good response to treatment in the caudal parts of the tumor with remaining viable tumor tissue in the cranial parts (Figure 3). The patient was scheduled for a second ablation procedure after 14 weeks to cover the formally not treated areas.

**Figure 4:**
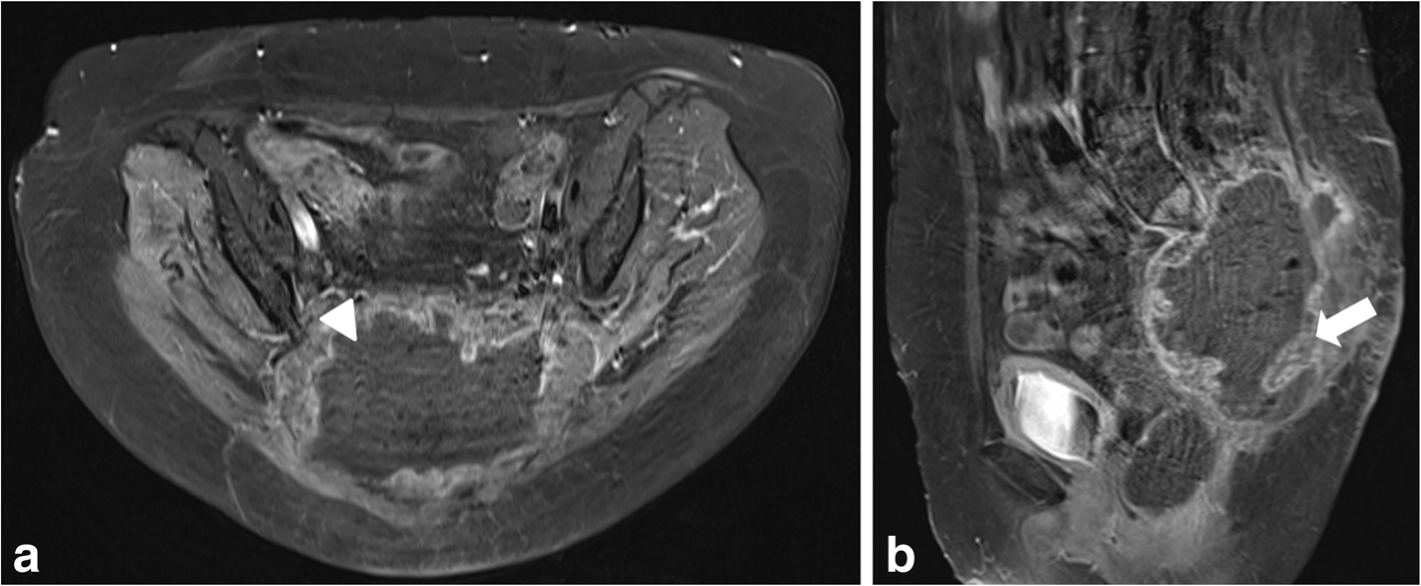
**Follow-up postcontrast T1-weighted magnetic resonance images at 8 weeks after second intervention.** Only peripheral parts of the tumor mass show viable enhancing tumor tissue (arrow). Central parts of the tumor are necrotic (arrow head). (**a**: axial plane, **b**: sagittal plane).

During the two IRE procedures the patient did not have any cardiovascular events, in particular no supraventricular tachycardia and no atrial fibrillation. Complications, especially postinterventional paralysis or bleeding, were not observed. After the first ablation procedure the patient did not complain about aggravated back pain; neither sensory deficit, nor loss of strength in her legs, nor paresthesia were observed. A neurological examination after the second ablation session revealed a mild 4+ paresis of the right extensor hallucis longus (L4 to S1) with complete resolution after 4 weeks. No sensory loss or impairment of bladder function occurred. After the second intervention opiate medication could be withdrawn. Using carbamazepine (200mg twice a day) and Polamidone (levomethadone) (5mg three times a day) pain control was achieved. The patient’s 24 hour follow-up imaging after the second ablation as well as follow-up imaging (Figure 4) after 8 weeks (consisting of contrast-enhanced ultrasound, MRI and a CT scan) showed wide ablation of the tumor with necrosis of most portions of the tumor and reduction of tumor volume to 791cm^3^.

## Discussion

Among the different tumor ablation techniques RFA is the most widespread technique [9]. Even though percutaneous ablation techniques are used as possibly curative therapies, palliative tumor ablation can be useful to achieve locoregional control of tumor growth, pain relief or pain control, especially in patients with unresectable tumor manifestations [10].

Due to heat dissipation to adjacent structures there is an inherent risk of thermal damage of adjacent organs, blood vessels and, of course, nerves. Thus, lesions close to adjacent structures with high risk of unintended heat destruction still pose a challenge for percutaneous thermal ablation techniques. IRE, in contrast to RFA, is a non-thermal ablation technique of soft tissue and offers a possibility to overcome the aforementioned limitations of thermal ablations. Instead of using heat, IRE uses a series of electrical pulses for microseconds to generate irreversible permeabilization of cell membranes, presumably through the formation of nanoscale defects in the cell membrane, and thereby induces cell death in the treated tissue.

IRE originally was viewed as an undesirable side effect of reversible electroporation and therefore was studied only to define the upper limit of electrical parameters that induce reversible electroporation. Due to its various features, for example transdermal delivery or introduction of drugs and genes into cells, or electrochemotherapy, reversible electroporation is an important method in biotechnology and medicine [11]. Davalos, Mir and Rubinsky in 2005 reported that IRE can be used as an independent modality for ablation of substantial tissue volumes [12]. Their findings were subsequently confirmed in experimental studies on cells and in large animal models [13,14].

In a series of studies, Lee could show that IRE produces irreversible tissue damage, which earlier was attributed only to thermal effects [15]. Furthermore, IRE proved to be especially effective in tissues with a high density of cell wall structures and less effective in tissues with a high concentration of collagenous and elastic fibers, which is suggestive of a cell selective effect [5,6]. There appears to be complete ablation up to the margin of blood vessels without compromising the functionality of the blood vessels [16]. This – in contrast to thermal ablation – would allow tumor cell ablation without concomitant destruction of connective tissue, blood vessels and nerves, which means ablation of tumor cells in those areas where thermal ablation was not possible before. In the proximity of larger blood vessels thermal ablation techniques are also hindered by the heat-sink effect. Due to its cooling effect blood flow is an important determinant as much as a limiting factor of thermal ablation techniques [17,18]. IRE seems to be unaffected by the blood flow and conversely does not potentially affect the macrovascularization of the ablation zone [19].

## Conclusion

Due to its more selective and non-thermal ablation effect IRE widens the field of minimally invasive treatable lesions. We showed in this case report that a large malignant lesion adjacent to a bundle of neural structures, that is the sacral plexus, can be widely ablated by IRE with only minor, temporary impairment of the neural function, even though a large infiltrating tissue volume was ablated.

## Consent

Written informed consent was obtained from the patient for publication of this case report and accompanying images. A copy of the written consent is available for review by the Editor-in-Chief of this journal.

## Competing interests

The authors declare that they have no competing interests.

## Authors’ contributions

PW, EMJ, BT, CN and CS performed the IRE procedure. AGS, JH, MR and WW were major contributors in writing the manuscript. All authors read and approved the final manuscript.
